# Human Cadaveric Model for Vessel Preparation Device Testing in Calcified Tibial Arteries

**DOI:** 10.1007/s12265-022-10319-9

**Published:** 2022-09-14

**Authors:** Bright Benfor, Kavya Sinha, Christof Karmonik, Alan B. Lumsden, Trisha L. Roy

**Affiliations:** 1 Department of Vascular Surgery, Houston Methodist Debakey Heart & Vascular Center, 6565 Fannin St, Suite B5-022, Houston, TX 77030 USA; 2 Translational Imaging Center, Houston Methodist Academic Institute, Houston, TX USA

**Keywords:** Tibial artery calcification, Arterial calcification, Peripheral vascular interventions, Vessel preparation, Artherectomy, Intravascular lithotripsy

## Abstract

To describe an ex vivo model for vessel preparation device testing in tibial arteries. We performed orbital atherectomy (OA), intravascular lithotripsy (IVL), and plain balloon angioplasty (POBA) on human amputated limbs with evidence of concentric tibial artery calcification. The arterial segments were then harvested for ex vivo processing which included imaging with microCT, decalcification, and histology. The model was tested out in 15 limbs and was successful in 14 but had to be aborted in 1/15 case due to inability to achieve wire access. A total of 22 lesions were treated with OA on 3/22 lesions, IVL on 8/22, and POBA without vessel preparation on the remaining 11/22. Luminal gain was assessed with intravascular ultrasound and histology was able to demonstrate plaque disruption, dissections, and cracks within the calcified lesions. A human cadaveric model using amputated limbs is a feasible, high-fidelity option for evaluating the performance of vessel preparation devices in calcified tibial arteries.

## Introduction

Peripheral arterial disease (PAD) is a growing global healthcare problem and a leading cause of major amputation of the lower extremities [1, 2]. The underlying pathophysiologic mechanisms are complex and involve endothelial dysfunction, accumulation of lipid in the vessel wall, inflammation, and vascular calcification [3]. The presence of calcium in tibial arteries is a common occurrence, particularly in patients with end-stage renal disease (ESRD) and diabetes mellitus [4]. While the etiology remains unclear, tibial arterial calcification (TAC) is associated with increased severity of PAD [5] and considered to be a major predictive factor of technical failure and postoperative complications of percutaneous interventions [4]. Kang and colleagues observed a technical failure rate of ~ 30% in patients with extensive TAC undergoing percutaneous vascular intervention (PVI) versus < 5% failure rate in those with minimal calcification [6]. These poor outcomes can be attributed to rigidity of the vessel wall that makes the blood vessel less compliant to balloon angioplasty. As a result, balloon angioplasty in calcified vessels leads to more uncontrolled dissections and limited luminal gain due to early recoil. Furthermore, the high balloon inflation pressures required to open thick calcified lesions may lead to intimal disruption and subsequent thromboembolic complications [4]. The great necessity to overcome these pitfalls and improve outcomes of PVI in calcified vessels has led to the emergence of several “vessel preparation” devices to debulk and modify calcified plaque and make it more amenable to balloon dilatation [7]. There are multiple commercially available atherectomy devices which are designed to mechanically debulk calcified lesions while leaving the internal elastic membrane intact, but these devices necessitate intimal disruption to remove plaque. On the other hand, intravascular lithotripsy devices have been designed to create micro-cracks within calcified plaques while sparing soft tissue and thus protecting the intima [7]. These devices were first employed in coronary vessels and later in peripheral arteries; however, there is little evidence to support their use in tibial arteries, and they continue to stir controversy due to the increased costs and potential for increased harm [8, 9]. Despite the controversies, there is an increasingly widespread use of these devices among vascular specialists. Given the associated financial cost and the possibility of their inappropriate use, it is important to perform robust studies to investigate their safety and efficacy. Randomized controlled trials (RCTs) offer a less-biased assessment of clinical endpoints related to PVI but do not evaluate the vessel wall response to percutaneous devices at the histologic level. The key difference between devices is the mechanism of action to restore blood flow while inducing minimal trauma to the vessel wall. To assess the benefit of one device over the other, this mechanistic data with histologic evaluation of the vessel wall is imperative to complement clinical RCTs. Current device testing is performed in animal models that are not reflective of the type and severity of calcified lesions that are encountered clinically [10]. In this study, we present the methodology and show the feasibility of a novel ex vivo model for vessel preparation device testing in tibial arteries using human amputated limbs.

## Methods

### Study Setting

This study was approved by the Institutional Review Board of the Houston Methodist Research Institute as part of a larger protocol evaluating the role of magnetic resonance imaging (MRI) in peripheral arterial disease management. Patients with planned above or below-knee amputation procedures for peripheral arterial disease (PAD) were contacted and informed consent obtained for participation in the study. The study sample consisted of fresh amputated limbs obtained from these patients.

### Eligibility Criteria

Pre-amputation imaging was studied, and limbs were excluded if there was no evidence of concentric calcification in any tibial artery. The focus of this study was concentric calcification as they have the highest rigidity and would demonstrate the most benefit of vessel preparation to modify compliance of the vessel. Concentric calcification was defined as a ≥ 270° calcification of the cross-section of the vessel wall as described by Fanelli [11]. In order to limit bias that can arise from prior revascularization procedures, amputated limbs with a known history of intervention on the tibial artery of interest were subsequently excluded from the study.

### Pre-interventional Handling and Imaging

**Fig. 1:**
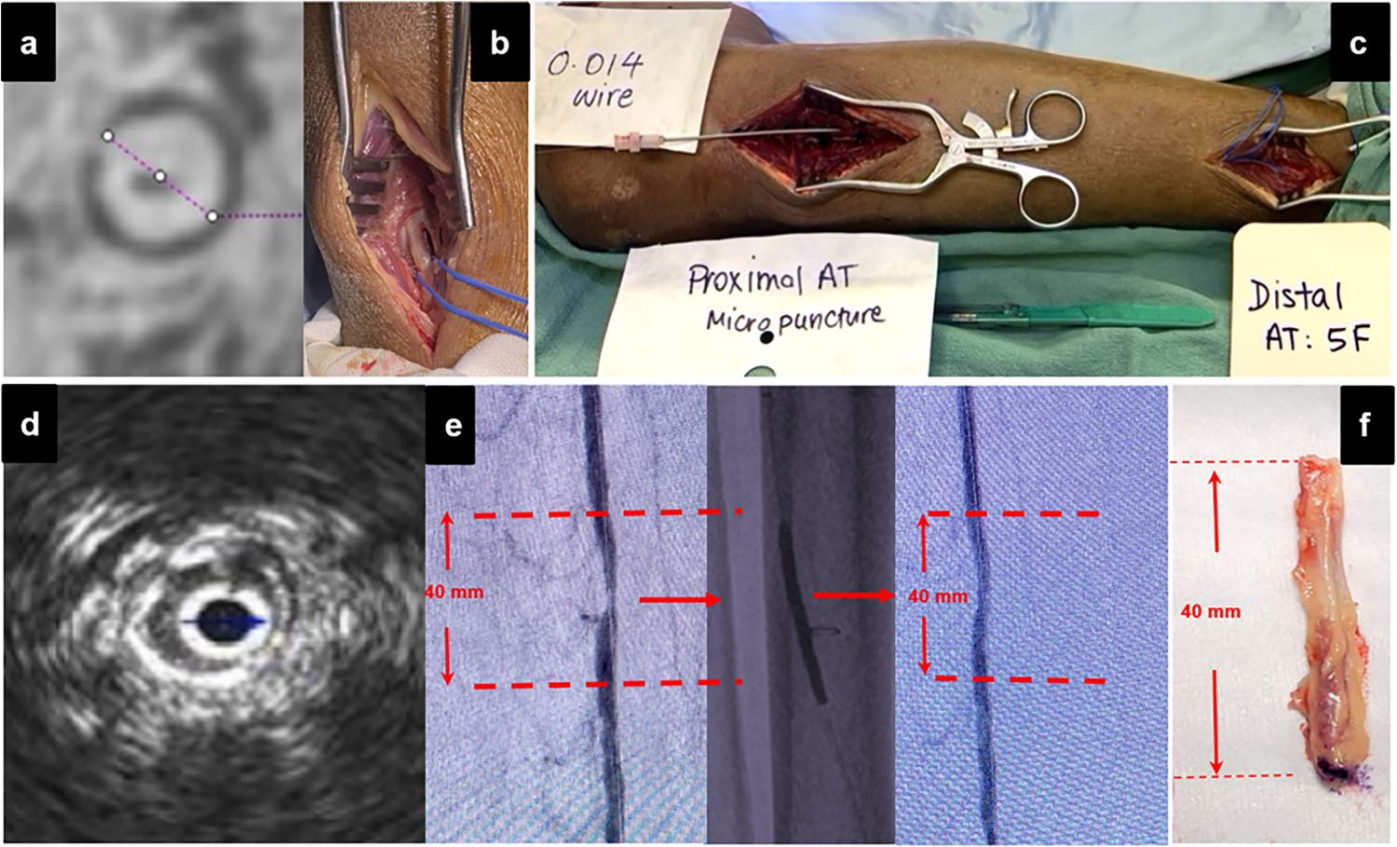
Experimental set up. **a** High-resolution MRI using ultrashort echo time sequences is used to characterize lesions at high resolution with the following parameters: 7 T: 28-channel clinical knee coil. UTE (FOV: 150 mm, TR 10 ms, TE 0.07 ms, flip angle 4). SSFP (FOV 160 mm, TR 12.57 ms, TE 6 ms, flip angle 25). The total acquisition time was 20 min (UTE, ultrashort echo time; SSFP, steady state free precession; FOV, field of view; TR, repetition time; TE, echo time). **b** The distal vessel was surgically exposed for arterial access under direct vision. **c** Amputated limb showing through and through 0.014″ wire access. **d** Intravascular ultrasound demonstrating concentric calcium in target lesion prior to intervention. **e** Angiography of the pre-intervention target lesion, angioplasty, and post-intervention completion angiogram. **f** Harvested target lesion for ex vivo analysis

Amputations were either below-knee (BKA) or above-knee (AKA) and performed for the appropriate clinical indication, and according to surgeon preference in either a traditional operating room or a hybrid OR. Vessels were tied off with silk ties during the amputation and blood within them remained until time of experiment when it was flushed out of the vessel with a pressurized bag of heparinized saline that infused throughout the procedure. The amputated limb was immediately transported from the OR to a translational imaging facility (Center for Translational Imaging at Houston Methodist Research Institute, Houston, TX) where it was imaged in an FDA-approved 7 T MRI scanner (MAGNETOM Serra ® Siemens Healthineers, Erlangen, Germany) at high resolution using ultra short echo time (UTE) and steady state free procession (SSFP). This imaging protocol, capable of differentiating between calcium and collagen, enabled histologic level lesion characterization of the native vessel before any wire or catheter was introduced into the lumen and potentially damaging the vessel wall (Fig. 1a). The distribution of calcification, reference vessel diameter and degree of stenoses as well as distance of the target lesion(s) from the medial malleolus on MRI were recorded for comparison with intraoperative measurements. Accurate co-registration with intraprocedural imaging was ensured by using the medial malleolus as a reference point to measure the distance to lesion location. The limb was then taken back into a hybrid suite for the experiment. The entire process from amputation to start of experiment lasted approximately ~ 2 h, including 1 h of MRI scan. Also, all pre-interventional handling of the limb as well as experiments were generally performed at room temperature (i.e., ~ 25C). On two occasions the limb had to be kept frozen overnight due to unavailability of the hybrid suite. The experiment was subsequently successful in one of those limbs.

### Vessel Preparation Device Description

**Fig. 2:**
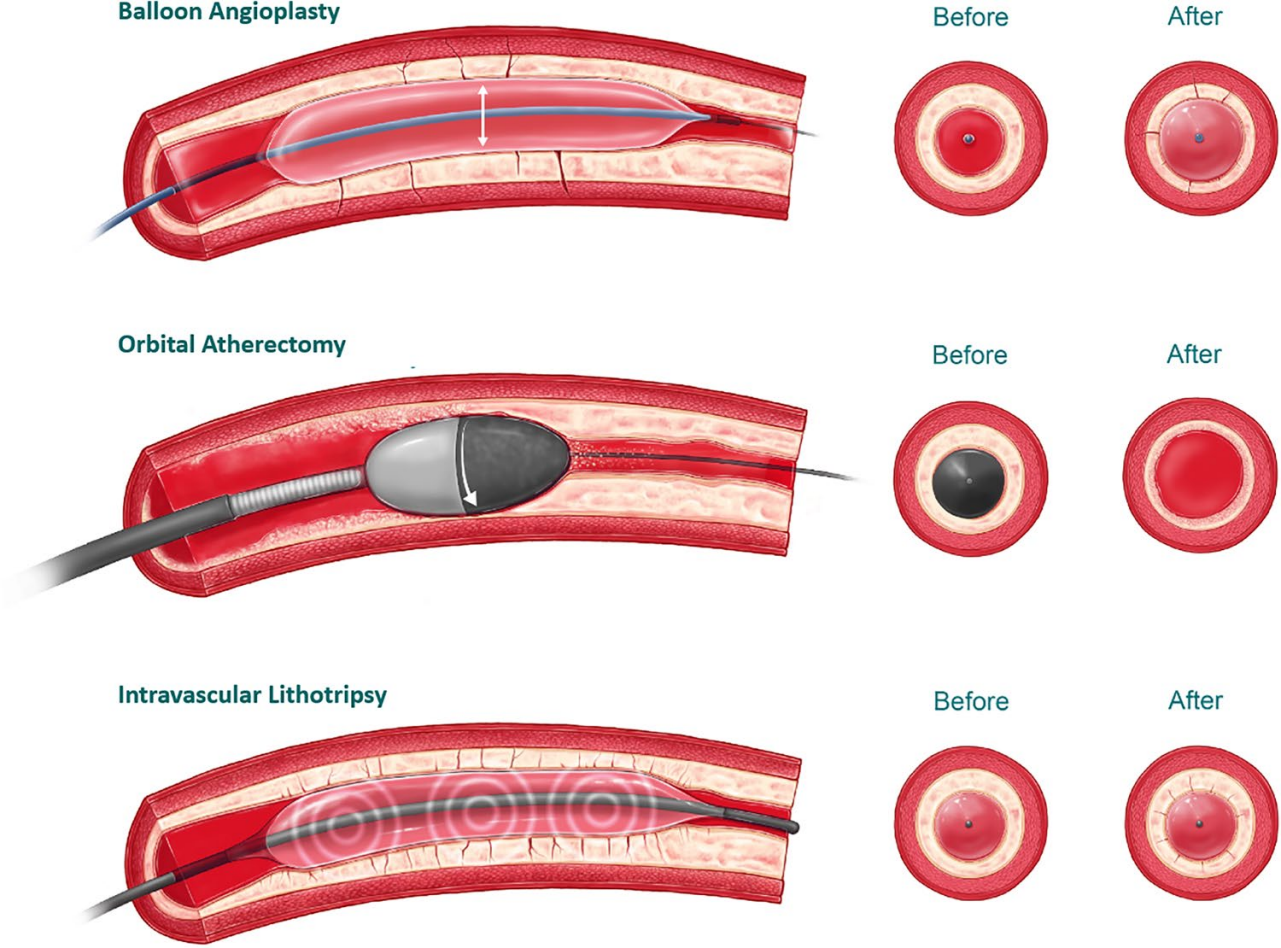
Illustration of the balloon angioplasty and vessel preparation techniques of calcified tibial arteries used in the experimental model

Two types of vessel preparation were investigated in this study (Fig. 2): orbital atherectomy (OA) with the DIAMONDBACK 360® Peripheral Orbital Atherectomy System (Cardiovascular System Inc., St. Paul, MI, USA) and intravascular lithotripsy (IVL) using the Shockwave S4 Peripheral Intravascular Lithotripsy catheter® (Shockwave Medical Inc., Santa Clara, CA, USA).

#### Diamond Back 360

The Diamond Back 360 (DB360) orbital atherectomy system has been previously described in the literature [12]. Briefly, the device consists of a handle and a cartridge with a drive shaft and an abrasive diamond-coated crown at the tip. The handle of the device is equipped with the electronic machinery and control buttons required to operate the rotational movements of the drive shaft. The system also comes with a saline line which connects the device to a saline pump for continual delivery of lubricant during the procedure.

#### Shockwave S^4^

The shockwave S4 system comprises a balloon catheter with integration of lithotripsy emitters capable of delivering localized pulses when the balloon is inflated. The system is also equipped with lithotripsy generator connected to the catheter by a connector cable [13]. The IVL catheter is compatible with a 5F sheath and has a working length of 135 cm and the balloon comes in four sizes: 2.5 × 40 mm, 3.0 × 40 mm, 3.5 × 40 mm, and 4.0 × 40 mm.

### Interventions

#### Obtaining Access

All procedures were performed in a hybrid operating room equipped with a robotic C-arm angiographic imaging system (Artis Pheno®, VE10, Siemens Healthineers, Germany).

The procedure began with surgical exposure of the distal tibial artery and intraluminal access was achieved with a micropuncture needle, 0.014 guide wire and a 5F sheath placed over the wire (Fig. 1). The wire was placed through and through, exiting the proximal end of the tibial artery which was transected to allow for flushing out blood clots with heparinized saline as well as contrast during the procedure. Infusion of heparinized saline was maintained throughout the procedure by connecting a pressurized saline infusion set to the side port of the 5F sheath using a three-way valve, and the liquid was collected — where the wire exited the body — in a container. After achieving access, an angiogram was then obtained to delineate the calcified stenotic lesions (Fig. 1e). The levels of the lesions were marked with radio-opaque staples on the skin and their distance from the medial malleolus recorded for accurate co-registration with preprocedural MRI. Angiographic diameters were recorded at the level of the lesions and at relatively healthy adjacent segments to calculate the degree of stenosis. The lesions were randomly assigned to receive either vessel preparation prior to plain old balloon angioplasty (POBA) or POBA alone. In the case of multiple lesions in the same vessel, they were considered separate lesions and treated differently (i.e., vessel preparation vs. POBA alone) if they were situated at least 4 cm apart. After this, an intravascular ultrasound (IVUS) was performed for lesion diameter measurements, confirmation of intraluminal access and balloon sizing. Once this was done, we proceeded to perform vessel preparation with chosen device according to manufacturer’s instruction for use.

For the purpose of developing this model, the choice of vessel preparation device (i.e., OA vs. IVL) depended on the availability of the device at the time of experiment.

#### Orbital Atherectomy with DB360

Upon intraluminal crossing of the lesion, a microcatheter was advanced and the guide wire was exchanged for an 0.014″ ViperWire® (Cardiovascular System Inc., St. Paul, MI, USA) which has been specifically designed for the DB360 device. We then proceeded to test the device to ensure the crown advancer knob (CAK) functioned properly before inserting into vessel. ViperSlide solution was continuously flushed through the device with a pressure bag. With the guidewire brake lever open, the CAK was locked at 1 cm and the device was gently advanced over the wire into the vessel under fluoroscopic guidance until the crown was positioned at approximately 1 cm beyond the lesion. The target lesion was then treated with a series of 30 s treatment intervals followed by rest periods of 30 s. An angiogram was performed to evaluate the reduction of stenosis and the rotational speed was gradually stepped up where necessary. Upon satisfactory angiogram, the spinning crown was stopped, the guidewire brake released, and the device gently retracted from the vessel while maintaining wire access. The orbital atherectomy system was then completely disconnected, and balloon angioplasty performed.

#### Intravascular Lithotripsy with Shockwave S^4^

The IVL balloon was first prepared by attaching a syringe filled with a 50/50 saline-contrast medium to the inflation port and pulling vacuum 3 times to replace air in the catheter with fluid. Next, the indeflator was filled with similar saline-contrast medium mixture and attached to the inflation port while ensuring no air was introduced into the balloon catheter. The guidewire port of the catheter, the shaft, and balloon were prepared with saline according to standard endovascular practice. With the IVL catheter connected to the pulse generator, the balloon was gently advanced over the wire and positioned at the treatment site. It was then inflated at 4 atm to treat the lesion, with an intermittent series of 20 pulses cycle and 10 s pause without exceeding 160 pulses. The balloon was inflated to nominal pressure (i.e., 6 atm) after each cycle, and the response of the vessel wall noted to see if additional pulses were required.

#### Plain Balloon Angioplasty

Balloon angioplasty was performed according to standard procedure. The size of the balloon was determined by the reference diameter per pre-interventional IVUS measurements however the length was maintained at 40 mm in keeping with the Shockwave IVL balloon length. Dilatation was performed at the minimum inflation pressure required to reach profile diameter and maintained for 3 min as prolonged inflations have been associated with reduced dissection rates [14]. Post-shockwave angioplasty was performed with the same balloon. The minimum pressure required for the angioplasty balloon to reach profile was recorded in each case for comparison.

#### Postinterventional Imaging

At the end of the intervention, final angiogram and IVUS were performed to measure and compare percentage of luminal gain, and to diagnose vessel wall dissection. After this all endovascular materials were retrieved from the vessel as we prepared to harvest the arterial segments. The entire experiment lasted ~ 2 h.

#### Ex Vivo Analysis

**Fig. 3:**
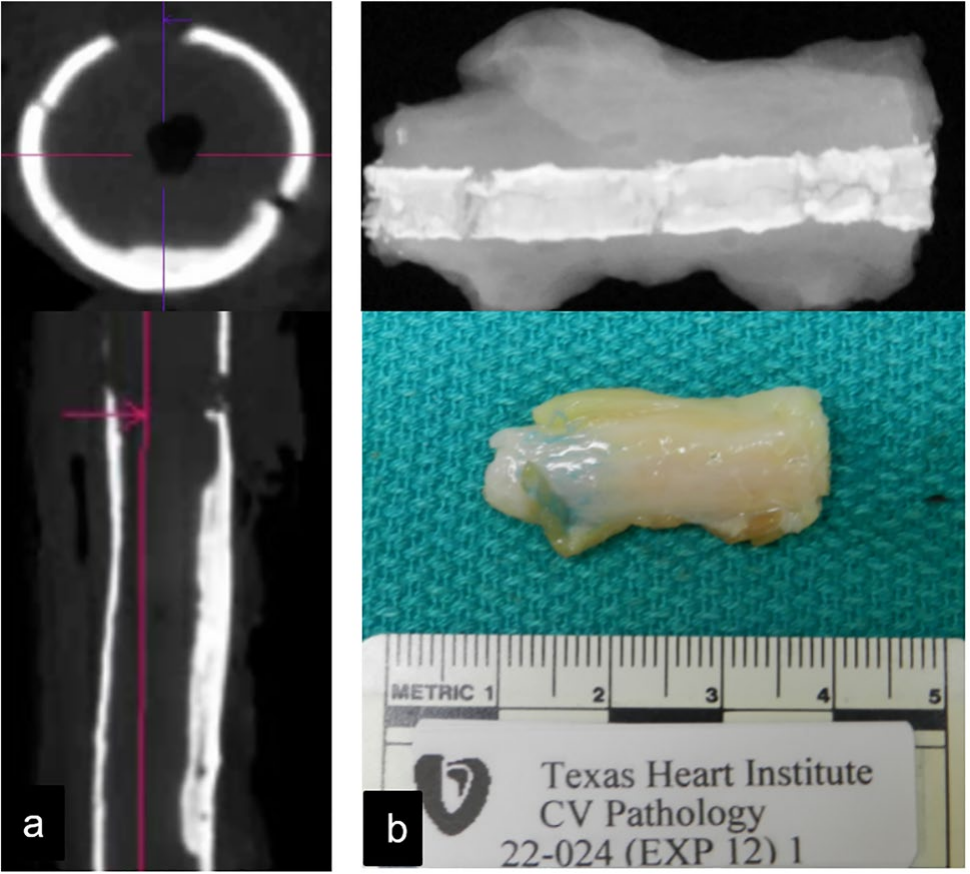
Ex vivo processing of arterial specimen. **a** MicroCT performed at high resolution for 3D characterization of calcium morphology. **b** Faxitron image and photograph of gross specimen of target lesion

The treated arterial segments were harvested by a vascular surgeon and/or senior trainee, segmented into 20 mm sections using an 11-blade scalpel, and stored in 4% percent formalin (Fig. 1f). Care was taken during this process to avoid fragmenting the calcified wall. A control portion of the vessel remote from the intervention site(s) was also harvested for comparison. The tissue samples were then taken for a same-day microCT imaging acquired at 40 μm × 40 μm × 40 μm, resolution to characterize areas of calcium prior to decalcification for histologic sectioning. After this, the samples were transferred to a cardiovascular histopathology laboratory (Texas Heart Institute, Houston, TX) where they were individually radiographed on a Faxitron MX-20 (Faxitron Bioptics LLC, Tucson Arizona), and decalcified with Cal-Rite (Richard Allen Scientific, Kalamazoo, MI, USA) when necessary (Fig. 3). Each arterial segment was serially sliced into approximately 5 mm-thick rings and two serial tissue Sects. (5-μm thick) were cut and stained with hematoxylin and eosin (H&E) and Movat pentachrome. Plaque components that were ambiguous were verified by a histopathologist from the Cardiovascular Pathology lab at Texas Heart Institute (Houston, Texas).

#### Outcomes Assessment

Luminal gain was assessed based on IVUS measurements, and defined as: $\frac{(Predilation\;degree\;of\;stenosis)-(Post\;dilatation\;degree\;of\;stenosis)}{Predilatation\;degree\;of\;stenosis}\mathrm{expressed}\;\mathrm{in}\;\mathrm{percentage}.$  

Balloon inflation pressure required to reach profile diameter was assessed as a secondary outcome measure, while histology assessed the degree of intimal disruption, dissections, and vessel wall damage.

## Results

**Fig. 4:**
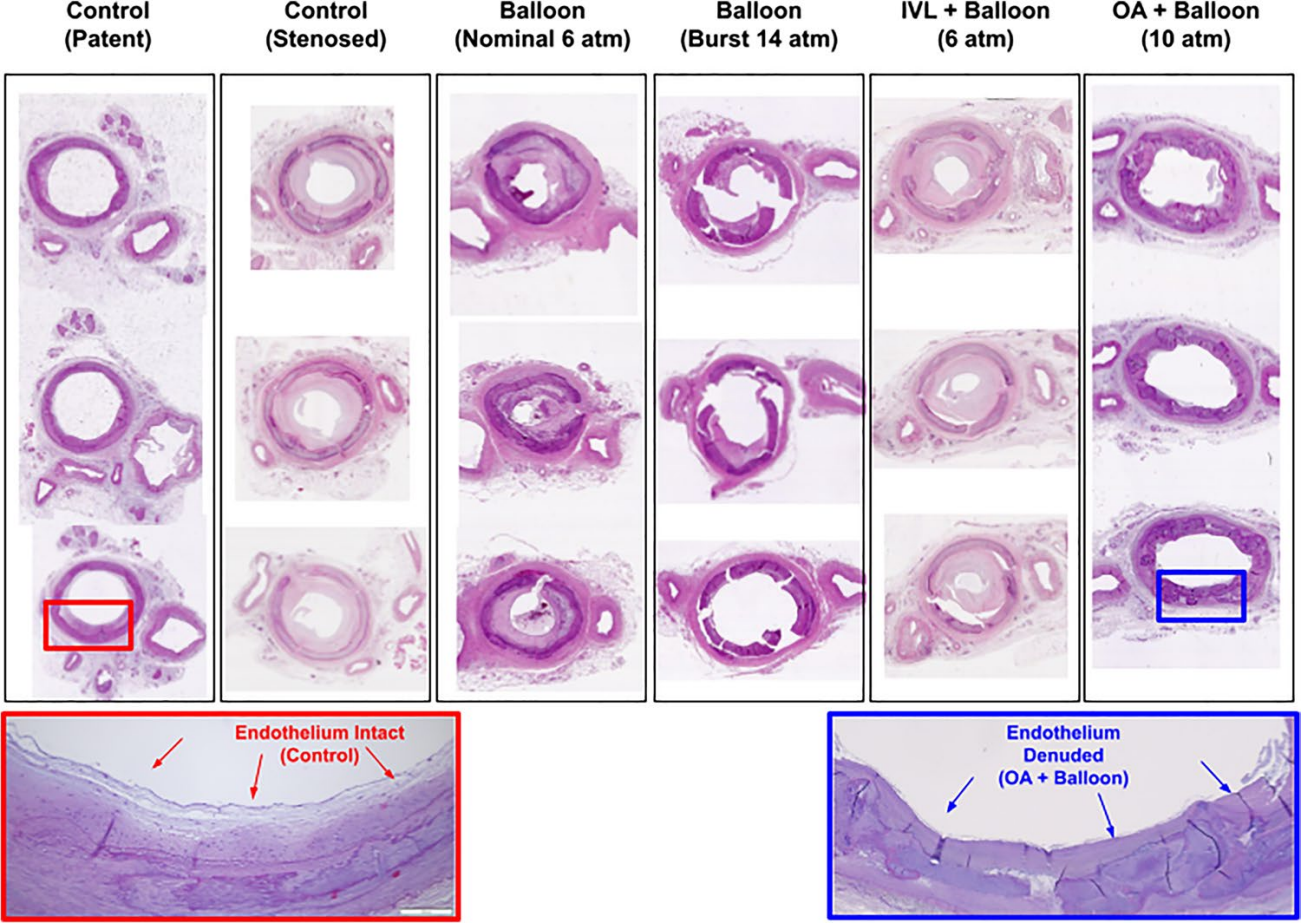
Histology of arterial segments showing the degree of intimal disruption and vessel wall damage with the various treatment modalities

The feasibility of this model was tested out in 15 amputated limbs: 6 below-the-knee amputations (BKA) and 7 above-the-knee amputations (AKA). Diabetes mellitus was present in 9/15 cases and end-stage renal disease in 9/15. The experiment was successfully carried out in 14/15 cases but had to be aborted in one due to inability to achieve intraluminal access (Table 1). The procedures were performed in the anterior tibial artery (ATA) in 11/15 cases, the posterior tibial artery in 3/15 cases and the peroneal in 1/15 limb, with a total of 22 lesions treated. Orbital atherectomy was performed on 3/22 lesions, IVL in 8/22 and POBA without vessel preparation in the remaining 11/22. The procedural details are presented on Table 2. The mean reference vessel diameter and average degree of stenosis were 2.9 ± 0.2 mm and 52 ± 6% respectively, while average luminal gain was estimated at 61 ± 27%. The average duration of the entire experiment was 120 ± 30 min. Typical histologic images of vessel wall based on type of treatment received are demonstrated in Fig. 4. We generally observed less luminal gain with minimal vessel wall damage at when POBA was performed at nominal pressure without vessel preparation, and greater luminal gain but excessive intimal disruption with uncontrolled dissections at burst pressures. On the other hand, orbital atherectomy denuded the endothelium but no uncontrolled dissections were observed. Finally, intravascular lithotripsy created some cracks within the calcium with minimal endothelial damage.

**Table 1:** Clinical characteristics of limbs used in amputation model for endovascular device testing in tibial arteries

Experiment	Age	Sex	Amputation	DM	ESRD	Target vessel	No of lesions	Treatment	Status of experiment
#1	82	F	AKA	Yes	Yes	ATA	1	POBA only	Completed
#2	79	F	AKA	Yes	Yes	ATA	1	POBA only	Completed
#3	47	F	BKA	Yes	Yes	ATA	2	OA /POBA only	Completed
#4	74	F	BKA	No	Yes	ATA	1	POBA only	Completed
#5	65	F	AKA	Yes	No	PTA	2	IVL/ POBA only	Completed
#6	84	F	AKA	No	No	ATA	1	IVL	Completed
#7	67	M	BKA	Yes	Yes	ATA	1	POBA only	Completed
#8	56	M	AKA	No	Yes	PTA	3	IVL/ IVL/POBA only	Completed
#9	59	F	BKA	Yes	Yes	ATA	3	IVL/ IVL/POBA only	Completed
#10	64	M	BKA	Yes	Yes	Peroneal	1	POBA only	Completed
#11	65	M	BKA	Yes	Yes	ATA	-	-	Aborted
#12	64	M	BKA	No	Yes	ATA	2	IVL/POBA only	Completed
#13	74	F	BKA	Yes	No	ATA	1	OA	Completed
#14	70	M	BKA	No	No	PTA	2	IVL/POBA only	Completed
#15	72	M	BKA	No	No	ATA	1	OA	Completed

*AKA* above-knee amputation, *ATA* anterior tibial artery, *BKA* below-knee amputation, *F* female, *M* male, *IVL* intravascular lithotripsy, *OA* orbital atherectomy, *PTA* posterior tibial artery, *POBA* plain old balloon angioplasty

**Table 2:** Procedural metrics of 14 successful experiments

Variable	Value
Total number of lesions	22
Orbital atherectomy, *n (%)*	3 (14)
Intravascular lithotripsy, *n (%)*	8 (36)
Balloon angioplasty only, *n (%)*	11 (50)
RVD, *mm, mean* ± *SD*	2.9 ± 0.2
Degree of stenosis, *%, mean* ± *SD*	52 ± 6
Acute luminal gain, %, *mean* ± *SD*	61 ± 27
Duration of procedure, *min, mean* ± *SD*	120 ± 30

*RVD* reference vessel diameter, *SD* standard deviation

## Discussion

In this study we demonstrated the feasibility of an ex vivo human cadaveric model for testing vessel preparation devices in tibial arteries. The need for high-fidelity experimental models to test the safety and efficacy of atherectomy and IVL devices is urgent given the paucity of clinical data to support their use in tibial arteries. Previous studies have shown favorable technical outcomes of atherectomy in tibial arteries but failed to demonstrate its superiority over POBA in infrapopliteal lesions [15, 16]. On the other hand, studies evaluating IVL in tibial arteries were largely non-comparative and therefore non-conclusive [13, 17]. Some authors have pointed at the methodology of these studies, as well as the lack of uniformity in lesion characterization, surgical indications, and definition of endpoints to explain the inconsistencies across studies, that make it difficult to draw conclusions on the comparative safety and efficacy of tibial vessel preparation [18]. The jury is therefore still out there, regarding the usefulness of vessel preparation in tibial arteries. We propose a robust and realistic human cadaveric model which has an advantage over animal models in that it directly reflects the type of calcified tibial lesions encountered in clinical practice. This model makes it possible to accurately quantify immediate luminal gain using IVUS, compare inflation pressures required to reach profile diameter, and evaluate vessel wall damage at the histology level. Concentric calcium was selected in our study to depict the type of lesions which stand to benefit the most from vessel preparation and therefore most likely to show difference in mechanistic performance compared to balloon angioplasty. On the contrary, if we are unable to demonstrate any differences at the histologic level of the vessel wall, then the likelihood of finding any clinical difference is very minimal. This model will therefore potentially complement clinical data in establishing the role of vessel preparation in tibial arteries. Furthermore, it enables head-to-head comparison of the different types of vessel preparation devices commercially available. Such studies are heavily lacking in the literature, but their findings would be very helpful in determining the right type of device for each type of lesion. The ex vivo analysis also make it possible to study the impact of different histological patterns of calcification on outcomes of endovascular interventions in tibial arteries [19]. In addition, apart from testing devices that are currently available commercially, this model will be potentially instrumental in preclinical testing of novel devices, given its high-fidelity compared to animal models. One other area in which this model could be useful will be drug-eluting technology in peripheral arteries. Some authors have previously argued that the presence of calcium diminishes the absorption of drugs in the vessel wall, thus rendering drug coated balloons and drug-eluting stents less effective in heavily calcified lesions [20]. In this regard, our model could be potentially useful in evaluating drug deposition and absorption in the arterial wall [21]. A major technical challenge with this model is the inability to achieve intraluminal wire access. The use of amputated limbs from live PAD patients suggests prior failed attempts at recanalization which could be reflective of the complexity of underlying disease. We therefore expected to see more technical failures with the experiments, however wire access was successful in all but 1/15 cases. We believe that careful selection of lesions based on pre-procedural imaging is key in achieving success with this model. This is where our preoperative MRI protocol excels, by differentiating between calcium and dense collagen which is more difficult to cross with wire [22]. Another difficulty is the accurate co-registration of preprocedural MRI, DSA and IVUS. We used the distance of lesions from the medial malleolus to aid in accurately lining up images. This could also be achieved by using vitamin E or fish oil capsules as skin markers during pre-interventional MRI and throughout the procedure, in addition to intraoperative lesion marking with radio-opaque staples [23, 24].

The lack of perfusion of tibial arteries may be considered a major limitation of this experimental model, rendering diameter measurements inaccurate as vessel walls are likely to collapse. In our experience however, this has not been the case. As demonstrated on preoperative MRI and ex vivo microCT, the presence of concentric calcium acts as a scaffold that tends to keep the arterial wall from collapsing and therefore does not need perfusion to keep its shape. We have compared our MRI images to pre-operative cross-sectional images that did not show significant differences in the caliber of the vessel. Furthermore, the arteries were distinct from the adjacent veins that collapse without perfusion.

## Conclusion

A human cadaveric model using amputated limbs is a feasible high-fidelity option for evaluating the mechanistic performance and safety of atherectomy and IVL in calcified infrapopliteal arteries. Non-invasive high-resolution MRI enables 3D plaque characterization prior to intervention to understand the mechanical impact of endovascular devices on the arterial wall. Furthermore, intraprocedural imaging complements the study to assess lesions immediately before and after intervention and finally ex vivo analysis allows the assessment of the vessel wall at the histologic level. This study demonstrated the feasibility of using this model and is now being implemented into a randomized study of vessel preparation devices for head-to-head comparisons to guide device selection in the future. The findings will complement clinical data in establishing the comparative outcomes of vessel preparation versus plain balloon angioplasty in the treatment of tibial artery calcification.

## Abbreviations

AKA: Above-the-knee
ATA: Anterior tibial artery
BKA: Below-the-knee
CAK: Crown advancer knob
CT: Computerized tomography
DB 360: Diamond Back 360
DSA: Digital subtraction angiography
ESRD: End-stage renal disease
FDA: Food and Drug Administration
FOV: Field of view
IEL: Internal elastic lamina
IVL: Intravascular lithotripsy
IVUS: Intravascular ultrasound
MRI: Magnetic resonance imaging
OA: Orbital atherectomy
PAD: Peripheral arterial disease
POBA: Plain old balloon angioplasty
PVI: Percutaneous vascular intervention
RCT: Randomized controlled trial
SSFP: Steady state free procession
TAC: Tibial artery calcification
TE: Echo time
TR: Repetition time
UTE: Ultrashort echo time
